# Working conditions and public health risks in slaughterhouses in western Kenya

**DOI:** 10.1186/s12889-016-3923-y

**Published:** 2017-01-05

**Authors:** Elizabeth Anne Jessie Cook, William Anson de Glanville, Lian Francesca Thomas, Samuel Kariuki, Barend Mark de Clare Bronsvoort, Eric Maurice Fèvre

**Affiliations:** 1 Centre for Immunity, Infection and Evolution, Institute for Immunology and Infection Research, School of Biological Sciences, University of Edinburgh, Ashworth Laboratories, West Mains Road, Edinburgh, EH9 3JT UK; 2 International Livestock Research Institute, Old Naivasha Road, PO Box 30709, 00100 Nairobi, Kenya; 3 Centre for Microbiology Research, Kenya Medical Research Institute, PO Box 19464–00200, Nairobi, Kenya; 4 The Roslin Institute, The Royal (Dick) School of Veterinary Studies, University of Edinburgh, Roslin, Midlothian, EH25 9RG UK; 5 The Royal (Dick) School of Veterinary Studies, University of Edinburgh, Roslin, Midlothian, EH25 9RG UK; 6 Institute of Infection and Global Health, University of Liverpool, Leahurst Campus, Chester High Road, Neston, CH64 7TE UK

**Keywords:** Slaughterhouse, Abattoir, Kenya, Food hygiene, Occupational safety

## Abstract

**Background:**

Inadequate facilities and hygiene at slaughterhouses can result in contamination of meat and occupational hazards to workers. The objectives of this study were to assess current conditions in slaughterhouses in western Kenya and the knowledge, and practices of the slaughterhouse workers toward hygiene and sanitation.

**Methods:**

Between February and October 2012 all consenting slaughterhouses in the study area were recruited. A standardised questionnaire relating to facilities and practices in the slaughterhouse was administered to the foreperson at each site. A second questionnaire was used to capture individual slaughterhouse workers’ knowledge, practices and recent health events.

**Results:**

A total of 738 slaughterhouse workers from 142 slaughterhouses completed questionnaires. Many slaughterhouses had poor infrastructure, 65% (95% CI 63–67%) had a roof, cement floor and walls, 60% (95% CI 57–62%) had a toilet and 20% (95% CI 18–22%) had hand-washing facilities. The meat inspector visited 90% (95% CI 92–95%) of slaughterhouses but antemortem inspection was practiced at only 7% (95% CI 6–8%). Nine percent (95% CI 7–10%) of slaughterhouses slaughtered sick animals. Only half of workers wore personal protective clothing - 53% (95% CI 51–55%) wore protective coats and 49% (95% CI 46–51%) wore rubber boots. Knowledge of zoonotic disease was low with only 31% (95% CI 29–33%) of workers aware that disease could be transmitted from animals.

**Conclusions:**

The current working conditions in slaughterhouses in western Kenya are not in line with the recommendations of the Meat Control Act of Kenya. Current facilities and practices may increase occupational exposure to disease or injury and contaminated meat may enter the consumer market. The findings of this study could enable the development of appropriate interventions to minimise public health risks. Initially, improvements need to be made to facilities and practices to improve worker safety and reduce the risk of food contamination. Simultaneously, training programmes should target workers and inspectors to improve awareness of the risks. In addition, education of health care workers should highlight the increased risks of injury and disease in slaughterhouse workers. Finally, enhanced surveillance, targeting slaughterhouse workers could be used to detect disease outbreaks. This “One Health” approach to disease surveillance is likely to benefit workers, producers and consumers.

**Electronic supplementary material:**

The online version of this article (doi:10.1186/s12889-016-3923-y) contains supplementary material, which is available to authorized users.

## Background

Slaughterhouses are defined as places where animals are slaughtered for food [1]. The development of the slaughter industry varies between countries due to cultural differences, the types of animals slaughtered and wealth [2]. In developed countries such as the USA or the United Kingdom traditional slaughter facilities were small and local to town centres [3, 4]. In the 20th century they became centralised, large-scale, and mechanized. They are now predominantly meat packing plants where animals are slaughtered and the meat is packed ready for distribution [3, 4]. One of the factors contributing to this change was supermarkets replacing butchers as the primary suppliers and the increase in restaurants and fast food establishments requiring large amounts of standardized products [3]. Large slaughter facilities had the necessary capital to respond to these market demands and also to the increased government regulations aimed at improving public safety both of which required upgrading equipment [3, 5].

In developing countries slaughter facilities vary from large industrial meat processing facilities in cities to small unregulated facilities in rural areas [6]. This variation in the meat industry is largely due to lack of private sector investment and inadequate regulation of the trade particularly in rural areas [7]. In addition there is often a deficit of suitable and/or affordable equipment for the processing and transportation of meat [7]. These factors combined with a lack of understanding of the risks of foodborne disease leads to poor conditions in rural slaughterhouses [7, 8].

Regulation of the slaughter industry aims to improve hygiene and reduce the contamination of meat and spread of disease, as well as protecting workers from occupational health hazards [7]. The meat industry in Kenya is regulated by the Directorate of Veterinary Services under the State Department of Livestock in the Ministry of Agriculture, Livestock and Fisheries [9]. A revised Meat Control Act was introduced in 2012 to standardise the meat industry across the country [9]. The revised Act provides information to reduce the risk of food borne disease and protect the consumer. The revised guidelines cover components of the slaughter process such as building structure and layout, equipment, personal hygiene, carcass handling, waste management, and meat inspection.

There are three types of slaughterhouses in Kenya depending on the size and whether the meat is for local consumption or transport out of the community. Slaughterhouses are further subdivided into ruminant or pig slaughterhouses, out of respect for the Muslim community. Category A slaughterhouses process over 40 bovines or greater than 8 pigs per day and are permitted to supply products all over Kenya. Category B slaughterhouses process 6–39 bovines or 1–7 pigs per day and are permitted to supply products up to 50 km from the slaughterhouse. Category C slaughterhouses process less than 5 bovines per day or less than 6 pigs and can only supply products to the local population centres.

Changes to slaughterhouses are now being implemented across Kenya to varying degrees. Introduction of the new regulations is slow in rural areas because abrupt enforcement may result in an increase in the informal market as local meat handlers are unwilling to meet the costs of the improved facilities [7, 8].

The majority of slaughterhouses in rural areas are classified under the new Meat Control Act as Category C, more commonly referred to as slaughter slabs. These facilities are privately owned and rented to butchers who employ their own team of slaughter workers [6, 8]. There is a smaller informal market for meat that continues outside the regulatory system that includes “backyard” slaughter [10]. Informal slaughter facilities are not regulated and may contribute to illegal livestock trading and the slaughter of diseased animals [6].

There are multiple failings in the slaughter process that result in meat contamination and allow the transmission of pathogens: inadequate infrastructure, poor hygiene, lack of ante and post mortem inspection, and inadequate training [8, 11]. Previous studies conducted in slaughterhouses in East Africa have highlighted the public health risks from food borne pathogens. Poor meat inspection has been indicated as contributing to the risks of bovine tuberculosis, toxoplasmosis and porcine cysticercosis [12–15]. Poor hygiene practices during carcass handling have been suggested as sources of meat contamination [16–18]. Lack of protective clothing has been identified as an occupational health risk for brucellosis in slaughterhouse workers in Uganda and Tanzania [19, 20]. Training and education for meat handlers and inspectors have been proposed by multiple authors investigating risks for food borne pathogens in the region [12, 16, 18]. Emerging zoonotic diseases, such as Rift Valley fever (RVF), have been reported in people involved in slaughter suggesting that slaughterhouses workers might be “sentinels” for disease emergence [21, 22].

There are no published reports describing the standards in slaughterhouses in western Kenya. This information is required to assess the potential risks to workers and consumers from these facilities and can be used to assess the impact of improvements. The present study reports on the facilities and hygiene practices in slaughterhouses in western Kenya as documented in 2012.

## Methods

### Study site

**Fig. 1:**
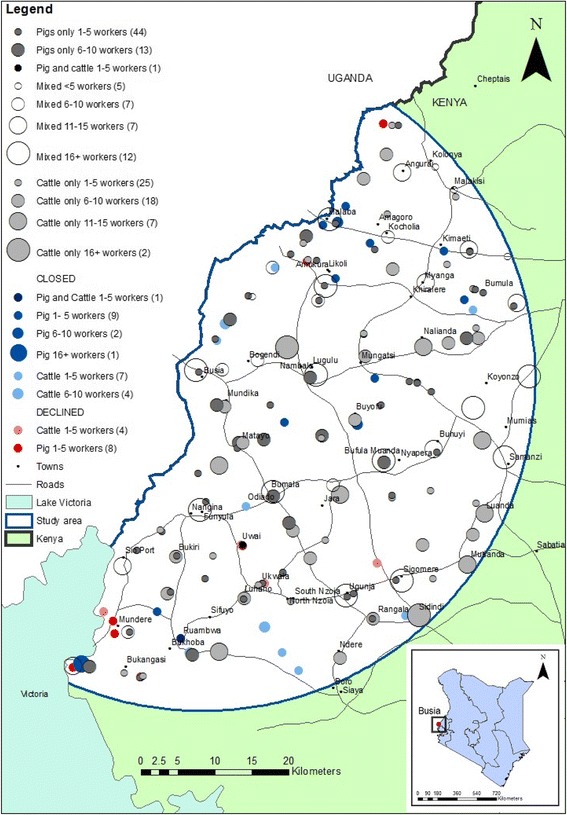
Map of slaughterhouses showing location, type and number of workers

The study was conducted in the Lake Victoria Basin region of western Kenya on the border with Uganda. The study area was a 45 km radius from Busia town, where the project laboratory is located (Fig. 1). It is a densely populated region with a population of 1.4 million people and a density of approximately 500 people per square kilometre (estimated from the Kenyan Human Population Census of 2009). The most common source of income is mixed subsistence farming [23] and it is estimated more than 40% of households are below the poverty line [24].

### Study population and recruitment

A census of slaughterhouses was performed between May 2011 and January 2012. The location of slaughterhouses in the study area was obtained from the former District Veterinary Officers (now County Directors of Veterinary Services) who had oversight over meat inspection. Data collection was conducted between February and October 2012.

### Sampling procedure

All slaughterhouses in the study area were visited 3–6 days before data collection. The purpose of this visit was to explain the project objectives and give the necessary information regarding the study so that participants could give informed consent. Participants were informed as a group by a project enumerator that they would be asked questions about their work and their health and samples would be tested for a range of diseases; it was emphasised that participation was voluntary. The biological sampling and data collection process was explained verbally. Workers were informed that they would receive a confidential report of any diagnoses and free treatment for any diagnosed condition.

On the day of data collection, informed consent was obtained from all participants individually. The enumerator outlined the objectives of the study as well as the questionnaire and biological sampling procedures. Participants were required to sign or apply a thumbprint to duplicate consent forms—one was retained and the other given to the participant. Inclusion criterion specified all workers, aged over 18 years and present at the slaughterhouse on the day of sampling. In slaughterhouses with 12 workers or less all willing participants were recruited. In slaughterhouses with greater than 12 workers a random selection of 12 willing participants from the workers present on the day were sampled. This restriction was necessary due to the time required to collect data each day, and also took into account that the slaughterhouses were professional environments where income earned by workers related to time worked.

Exclusion criteria included third trimester pregnancy (self reported), severe anaemia (assessed by mucous membrane pallor), being under the age of eighteen (self-reported), severe inebriation (determined if the participant was unable to converse clearly without slurred speech or confusion), aggression toward the project enumerator, and being over eighty-five years (self-reported).

### Data collection

Three data collection tools were used to obtain data regarding slaughterhouses and workers.

- A 114-item individual questionnaire (Additional file 1) was administered to each participant by one of seven trained interviewers. Interviews were conducted in Kiswahili, Dholuo, Luhya and English depending on the language in which the participant was most comfortable. Data were collected on personal history (such as age, gender, marital status and education), dietary habits, knowledge of zoonoses, risk behaviours, exposure to livestock, and personal hygiene practices at the slaughterhouse. The interviewer also recorded if the participant appeared to have consumed alcohol.
- A second 72-item questionnaire (Additional file 2) was administered to the foreperson of the slaughterhouse regarding slaughterhouse structure, equipment, and practices.
- The interviewer recorded observations regarding facilities and practices where slaughtering was observed at the time of interview. The observations were recorded by the enumerator after completion of the foreperson interview. Observations were recorded as present or absent on a standardised template. These included the presence of: the meat inspector at the slaughterhouse and if he/she conducted antemortem inspection; a latrine within the compound; designated handwashing facilities, and soap; a pit to dispose of carcass waste; dogs around the slaughterhouse; if workers wore protective clothing/boots and were seen eating.

Questionnaires were pretested in 3 slaughterhouses bordering the study area through January 2012. Questionnaire and observation data were recorded on a Palm operating system (Palm OS) Personal digital assistant (PDA) using Pendragon Forms 5.1 (Pendragon Software Corporation, Libertyville, IL). Microsoft® Access databases were used to manage data.

### Mapping

Slaughterhouses were georeferenced using a handheld Global Positioning System (GPS) device (Garmin eTrex®). The locations of slaughterhouses were mapped using ArcGIS™ version 9.1 and version 10.2.2 (ESRI, Redlands, California, USA). Base layers were provided by the ILRI geographical information systems unit (http://www.ilri.org/gis).

### Data analysis

Descriptive statistics were performed in R software version 3.0.2 [25]. The *Survey* package [26] in R was used to adjust for the complex survey design. Weights for the slaughterhouse level data were calculated by dividing the number of each type of slaughterhouse by the number sampled. Weights for the slaughterhouse worker data were calculated by dividing the total number of slaughterhouse workers in the slaughterhouse by the number sampled, with slaughterhouse used as a clustering variable. Design-based adjustment was implemented using the *svydesign* procedure in the *Survey* package in R [27].

Variables were analysed for independence using the *svychisq* command in *Survey* which calculated a Pearson’s Chi squared statistic adjusted by the complex design. A level of 5% statistical significance (Type 1 error) was used.

## Results

There were 180 slaughterhouses in the study area when the study began in May 2011. Twenty-four slaughterhouses were closed by the former District Veterinary Officers (now County Directors of Veterinary Services) between May 2011 and January 2012 for non-compliance with regulations. From the remaining 156, 142 slaughterhouses (91%) agreed to participate in the study with fourteen (9%) slaughterhouses refusing to participate. This included 4/57 (7%) cattle and 10/68 (15%) pig slaughterhouses. Although no specific reason was given for refusal, the study team surmised that fear of recriminations from the Department of Veterinary Services was an important factor.

Figure 1 shows the distribution of slaughterhouses in the study area. There was one cattle and one pig slaughterhouse that used the same facility but for the purposes of analysis, they were considered separate slaughterhouses as the workers were different. The slaughterhouses were evenly distributed throughout the study area. The slaughterhouses that refused to participate appeared to be clustered in the south of the study area.

Of the 142 slaughterhouses recruited in the study, 31 were mixed ruminant (cattle, goats, sheep), 53 were cattle only and 58 were pig only. The total employment at these slaughterhouses was 1005 workers. Questionnaires were administered to 738 (73.4%) workers at all 142 slaughterhouses. Slaughtering was observed at 84 slaughterhouses whilst interviews were being conducted.

The results are presented in two parts. The questionnaires completed by the foreperson together with the observations by the study team form the results regarding the slaughterhouse infrastructure and practices. The second questionnaires administered to the workers and the clinical assessment of the health of those workers form the results regarding worker practices, knowledge, and health.

### Slaughterhouse infrastructure and practices

Only two slaughterhouses were Category B slaughterhouses. The remainder were Category C or informal. It was not possible to determine which slaughterhouses were working without authority and would therefore be classified as informal slaughterhouses, as forepersons were unwilling to admit to working outside the regulations.

**Fig. 2:**
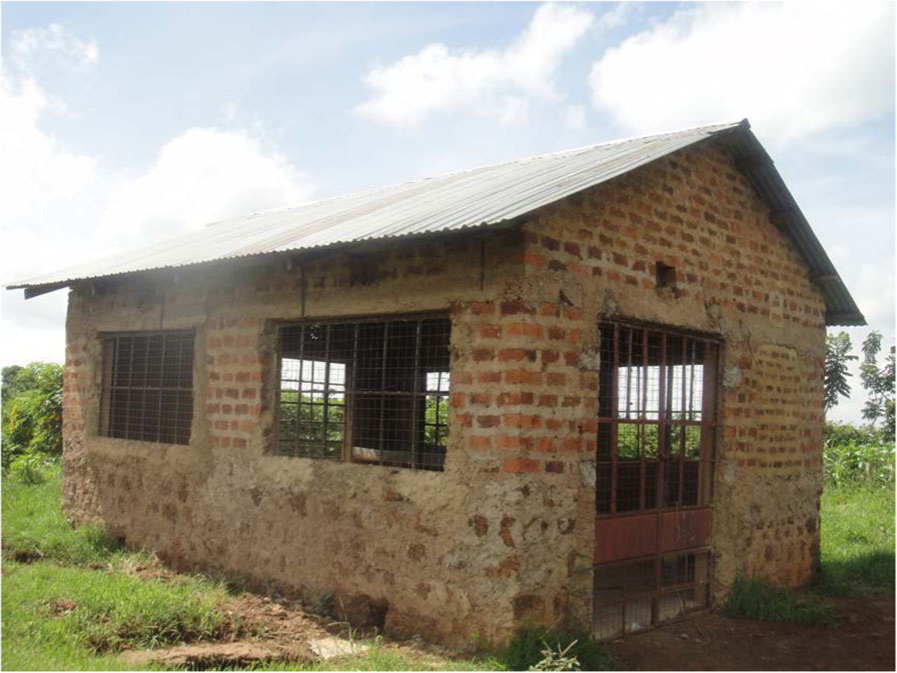
External appearance of a category C cattle slaughterhouse in western Kenya

**Fig. 3:**
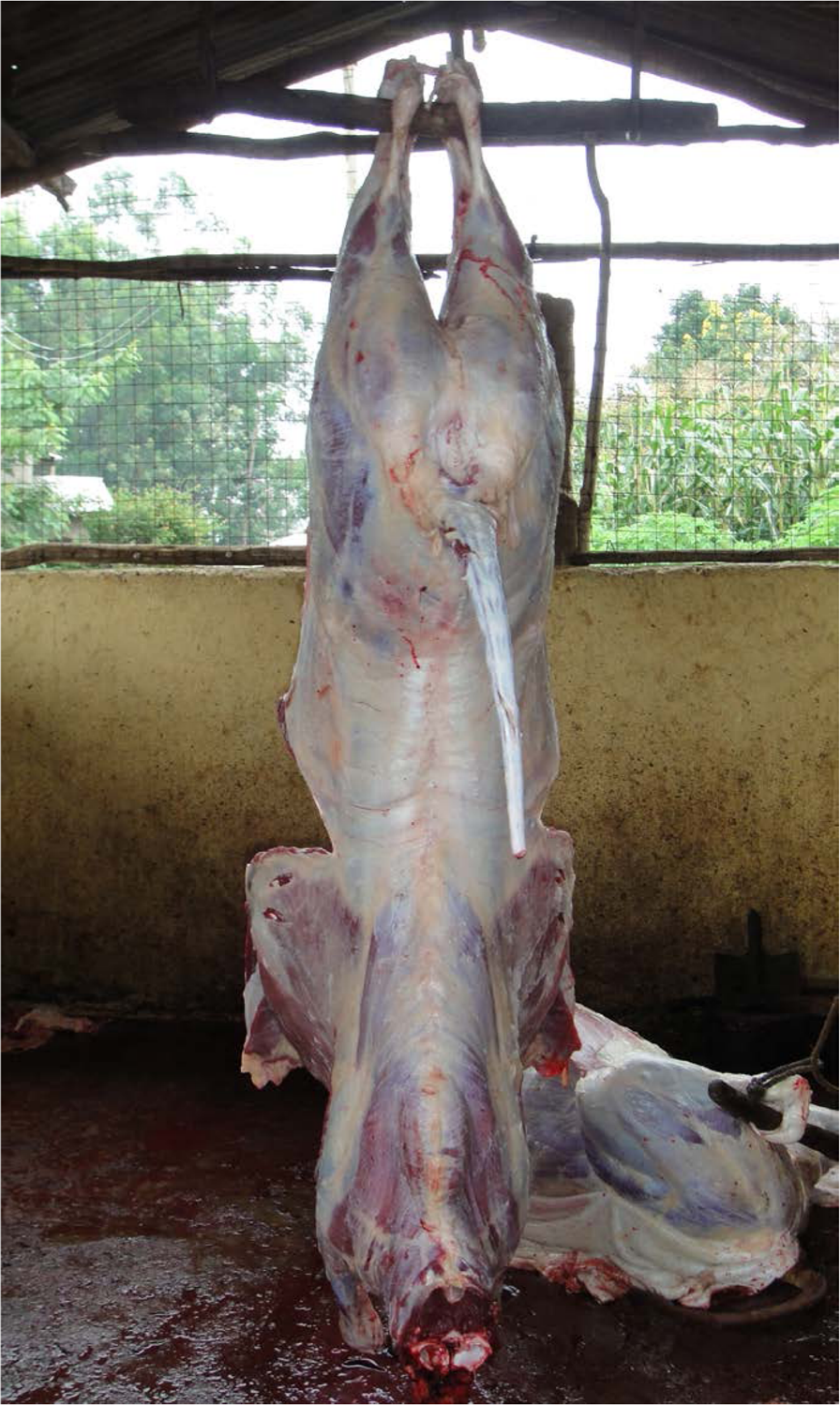
Internal appearance of a category C cattle slaughterhouse in western Kenya

Despite the vast majority of slaughterhouses being classed as Category C, only 26% (95% CI 24–28) restricted meat selling within the local village, with the remainder transporting meat outside the immediate area. Figures 2 and 3 demonstrate the external and internal appearance (respectively) of Category C cattle slaughterhouses in western Kenya.

Table 1 details the results of the questionnaire and Table 2 the observations regarding the infrastructure and practices at slaughterhouses. Only 65% (95% CI 63–67%) of slaughterhouses had a roof, cement floor, and solid walls. There was a general lack of electricity and piped water, with 3% (95% CI 3–4%) of all slaughterhouses having either utility. The majority of slaughterhouses, 66% (95% CI 64–68%), sourced water from boreholes (deep vertical holes drilled in the earth) and water was carried by hand to the slaughterhouse. There was a lack of sanitation facilities with only 60% (95% CI 57–62%) of slabs reporting to have latrines and 20% (95% CI 18–22%) to have hand washing facilities. These reports were corroborated by the interviewer observations that 60% had toilets (95% CI 52–67%) and 12% (95% CI 7–16%) had hand-washing facilities. A large number of slaughterhouses (78%; 95% CI 76–80%) reported seeing dogs around the facility with smaller percentage seeing rats (12%; 95% CI 11–14%). A pit for carcass waste was observed at the majority of slaughterhouses (93%; 95% CI 89–97%).

**Table 1:** Facilities and hygiene practices in slaughterhouses

Variable	Mixed Ruminant % *n* = 31	Cattle % (95%CI) *n* = 53	Pig only % (95%CI) *n* = 58	Total % (95%CI) *n* = 142	Chi squared, *p* –value
Structural factors					
Roof present	90	75 (72–79)	45 (40–50)	65 (63–67)	X^2^ = 21.53, df = 2 *p* < 0.001
Cement floor	100	100	74 (70–78)	89 (87–90)	X^2^ = 23.39, df = 2 *p* < 0.001
Solid walls	97	79 (76–82)	53 (48–58)	72 (69–74)	X^2^ = 20.25, df = 2 *p* < 0.001
Electricity	3	0	2 (0.4–3)	1.4 (1–2)	X^2^ = 1.48, df = 2 *p* < 0.001
Sanitation					
Toilet	61	57 (53–60)	62 (57–67)	60 (57–62)	X^2^ = 0.38, df = 2 *p* = 0.117
Piped water	6	6 (4–7)	0	3 (3–4)	X^2^ = 3.82, df = 2 *p* < 0.001
Hand-washing place	35	19 (16–22)	14 (10–17)	20 (18–22)	X^2^ = 5.76, df = 2 *p* = <0.001
Cleaned with soap	90	83 (80–86)	62 (57–67)	75 (73–78)	X^2^ = 10.92, df = 2 *p* < 0.001
Dogs present	71	74 (70–78)	85 (81–88)	78 (76–80)	X^2^ = 2.89, df = 2 *p* < 0.001
Rats present	10	6 (4–7)	19 (15–23)	12 (11–14)	X^2^ = 4.87, df = 2 *p* < 0.001
Source of water					
Borehole	65	70 (67–73)	64 (59–69)	66 (64–68)	X^2^ = 3.83, df = 2 *p* < 0.001
Municipal	13	13 (11–16)	7 (4–9)	10 (9–12)	
River	3	8 (6–9)	10 (7–13)	8 (7–9)	
Well	19	9 (7–12)	19 (15–23)	16 (14–17)	
Personal hygiene					
Protective clothing worn	55	36 (32–39)	17 (13–21)	32 (29–34)	X^2^ = 13.38, df = 2 *p* < 0.001
Worker buys clothing	90 (87–93)	78 (71–85)	67 (51–82)	78 (73–84)	
Boots worn	52	45 (41–48)	16 (12–19)	34 (31–36)	X^2^ = 16.33, df = 2 *p* < 0.001
Worker buys boots	92 (87–97)	84 (77–91)	72 (54–90)	84 (78–89	
Soap provided	81	64 (61–68)	57 (52–62)	64 (62–67)	X^2^ = 4.75, df = 2 *p* < 0.001
Meat inspection					
Meat inspector visits daily	100	100	84 (81–88)	93 (92–95)	X^2^ = 13.36, df = 2 *p* < 0.001
Antemortem exam	13	6 (4–7)	5 (3–7)	7 (6–8)	X^2^ = 1.99, df = 2 *p* < 0.001
Slaughter a sick animal	19	8 (6–9)	5 (3–7)	9 (7–10)	X^2^ = 3.69, df = 2 *p* < 0.001
Meat exported					
Meat sold only to local village	10	30 (27–33)	29 (25–34)	26 (24–28)	
Meat exported from sublocation	26	19 (16–22)	19 (15–23)	20 (18–22)

**Table 2:** Structure and practices of the slaughterhouses as observed by the interviewer

	Mixed % (95%CI) *n =* 28	Cattle % (95%CI) *n =* 31	Pigs only % (95%CI) *n =* 25	Total % (95%CI) *n =* 84
Sanitation				
Pit	100	100	84 (72–96)	93 (89–97)
Toilet	57 (51–63)	65 (53–76)	56 (40–72)	60 (52–67)
Hand washing place	32 (27–38)	10 (3–17)	4 (2–10)	12 (7–16)
Dogs present	64 (59–70)	97 (93–100)	80 (67–93)	83 (77–89)
Personal hygiene				
Protective clothing worn >50% workers	64 (59–70)	35 (24–47)	4 (0–10)	27 (21–34)
Boots worn by >50% workers	57 (51–63)	26 (15–36)	4 (0–10)	22 (17–28)
Soap provided	50 (44–56)	16 (7–25)	12 (2–22)	21 (15–27)
Eating observed	18 (13–22)	6 (1–12)	28 (14–42)	18 (12–24)
Meat inspection				
Meat inspector seen	79 (74–83)	65 (53–76)	32 (17–47)	53 (45–61)
Antemortem inspection	14 (10–18)	10 (3–17)	0	6 (3–10)

Both mixed ruminant slaughterhouses and cattle only slaughterhouses had better infrastructure than pig slaughterhouses. Ninety percent of mixed ruminant slaughterhouses and 75% (95% CI 72–79%) of cattle only slaughterhouses had a roof, cement floor and solid walls compared with 45% (95% CI 40–50%) of pig slaughterhouses (X^2^ = 21.53, df = 2, *p <* 0.001).

Slaughtering, bleeding, skinning, and evisceration were performed in the same area in all slaughterhouses (batch slaughtering). The viscera were washed outside the slaughterhouse on a concrete slab in all but one slaughterhouse, where there was a specific room inside the slaughterhouse. Only one mixed ruminant slaughterhouse stunned cattle before slaughter. The remaining 141 slaughterhouses cut the throat without prior stunning.

Less than half of slaughterhouses reported that workers wore personal protective clothing. The forepersons in 32% (95% CI 29–34%) of slaughterhouses reported that workers wore protective coats; and in 34% (95% CI 31–36%) that workers wore rubber boots. This report was supported by the observational data that workers in 27% (95% CI 21–34%) of slaughterhouses wore lab coats and workers in 22% (95% CI 17–28%) of slaughterhouses wore rubber boots.

Very few slaughterhouses provided protective equipment for workers, with workers providing their own protective coats in 78% (95% CI 73–84%) of slaughterhouses and workers providing their own rubber boots in 84% (95% CI 78–89%) of slaughterhouses. No workers were observed wearing gloves. Workers in mixed ruminant (55%) and cattle slaughterhouses (36%; 95% CI 32–39%) were more likely to wear protective coats than workers in pig slaughterhouses (17%; 95% CI 13–21%) (X^2^ = 13.38, df = 2 *p =* 0.001). Workers in mixed ruminant slaughterhouses (52%) and cattle slaughterhouses (45%; 95% 41–48%) were more likely to wear boots than workers in pig slaughterhouses (16%; 95% CI 12–19%) (X^2^ = 16.33, df = 2 *p <* 0.001).

Soap was reported to be provided at 64% (95% CI 62–67%) of slaughterhouses but was only observed in 21% (95% CI 15–27%). Soap was observed in 16% (95% CI 7–25%) of cattle only and 12% (95% CI 2–22%) of pig only slaughterhouses. This was significantly less than in mixed ruminant slaughterhouses where soap was observed 50% (95% CI 44–56%) of the time (X^2^ = 4.75, df = 2 *p =* 0.001). Eating was observed in 18% (95% CI 12–24%) of slaughterhouses.

Ninety percent (95% CI 92–95%) of slaughterhouses reported that the meat inspector visited every day. However the meat inspector was seen at only 53% (95% CI 45–61%) of slaughterhouses during the course of data collection. Workers explained that the meat inspector may visit the butchery to inspect the meat if he/she was too late arriving and did not see the meat at the slaughterhouse. Antemortem inspection was reported at 7% (95% CI 6–8%) and observed at 6% (95% CI 3–10%) of slaughterhouses. Nine percent (95% CI 7–10%) of slaughterhouses reported slaughtering animals they identified as being sick/ill/unhealthy.

### Slaughterhouse worker practices, knowledge and health

The slaughterhouse workers ranged in age from 18–82 years with a mean age of 39 (95% CI 39–40). The mean time of employment as a slaughterhouse worker was 9.35 years (95% CI 9–10) with a range of 1 month to 59 years. The mean days worked per week were 4.9 with a mean work day of 2.5 h.

The different jobs in the slaughterhouses included slaughtermen (11%; 95% CI 9–14%); flayers (75%; 95% CI 72–78%); cleaners (4%; 95% CI 4–5%); the person who cleaned the offal (8%; 95% CI 6–10%); and foreperson/owner (2%; 95% CI 1–3%). The slaughterman is responsible for cutting the animals throats in mixed ruminant and cattle slaughterhouses. The slaughterman is a practicing Muslim so that all meat products are Halal. Flayers are responsible for skinning and sectioning the carcass. There is not an official slaughterman in pig slaughterhouses. The same worker that cuts the throat also sections the carcass and in this study these people are classified as flayers. In ruminant and cattle slaughterhouses a specific worker is responsible for washing the offal to remove gross faecal contamination as offals are sold for consumption. Pig offals are not typically consumed in the study area, and these were not observed to be processed.

Ninety-seven percent (95% CI 96–97%) of slaughterhouse workers were men. Seventy-four percent (95% CI 73–76%) of workers had primary level education. Eighty-two percent (95% CI 80–83%) of workers had a second occupation, predominantly as butchers (42%; 95% CI 40–44%) or farmers (28%; 95% CI 27–30%). Seventy-two percent (95% CI 70–74%) of workers had contact with livestock outside of work. The majority of workers had contact with poultry (88%; 95% CI 86–89%) and cattle (72%; 95% CI 70–74%). Contact with other animals included goats (42%; 95% CI 40–44%); sheep (25%; 95% CI 24–47%); and pigs (37%; 95% CI 35–39%).

Table 3 details the results of the questionnaire regarding the personal hygiene practices at slaughterhouses. Fifty-three percent (95% CI 51–55%) of workers reported wearing protective clothing. Workers at ruminant slaughterhouses (69%; 95% CI 66–73%) and cattle only slaughterhouses (49%; 95% CI 46–51%) were more likely to wear protective clothing compared with pig slaughterhouse workers (27%; 95% CI 23–30%) (X^2^ = 79.82, df = 2 *p <* 0.001). Forty-nine percent (95% CI 46–51%) of workers reported wearing rubber boots. Workers at ruminant slaughterhouses (68%; 95% CI 64–71%) and cattle slaughterhouses (41%; 95% CI 38–44%) were more likely to wear rubber boots compared with pig slaughterhouse workers (22%; 95% CI 19–26%) (X^2^ = 95.14, df = 2 *p <* 0.001) (Table 3).

**Table 3:** Personal hygiene practices and knowledge in slaughterhouses

Variable	Mixed % (95%CI) *n =* 274	Cattle % (95%CI) *n =* 292	Pigs only % (95%CI) *n =* 172	Total % (95%CI) *n =* 738	Chi squared
Personal hygiene					
Wear protective clothing	69 (66–73)	49 (46–51)	27 (23–30)	53 (51–55)	X^2^ = 76.41, df = 2 *p <* 0.001
Wear boots	68 (64–71)	41 (38–44)	22 (19–26)	49 (46–51)	X^2^ = 94.94, df = 2 *p <* 0.001
Soap available	50 (46–54)	62 (59–65)	68 (64–72)	58 (56–60)	X^2^ = 16.03, df = 2 *p <* 0.001
Eat at the slaughterhouse	27 (23–30)	5 (5–7)	37 (33–41)	21 (20–23)	X^2^ = 78.88, df = 2 *p <* 0.001
Smoke daily	22 (19–25)	27 (24–29)	19 (16–22)	23 (21–25)	X^2^ = 4.74, df = 2 *p =* 0.09
Take alcohol daily	33 (30–37)	31 (28–33)	32 (28–36)	32 (30–34)	X^2^ = 0.27, df = 2 *p =* 0.87
Use the latrine regularly	73 (70–76)	78 (75–80)	78 (75–82)	76 (74–78)	X^2^ = 2.4, df = 2 *p =* 0.30
Meat inspection					
Meat inspector visits	98 (97–99)	99 (99–100)	84 (81–87)	96 (95–96)	X^2^ = 60.17, df = 2 *p <* 0.001
Antemortem exam	44 (41–48)	48 (45–51)	34 (30–38)	44 (42–46)	X^2^ = 9.02, df = 2 *p =* 0.01
Slaughter sick animal	23 (19–26)	14 (12–15)	15 (12–18)	18 (16–19)	X^2^ = 8.77, df = 2 *p =* 0.01
Zoonoses awareness					
Know animals give disease to people	34 (31–38)	30 (27–32)	29 (25–33)	31 (29–33)	X^2^ = 1359, df = 2 *p =* 0.45
Know disease can be transmitted from meat	45 (41–49)	38 (35–40)	42 (38–46)	42 (40–44)	X^2^ = 2.77, df = 2 *p =* 0.25
Named a zoonosis	8 (6–10)	8 (6–10)	9 (6–11)	8 (7–9)	X^2^ = 0.11, df = 2 *p =* 0.95
Named a disease from meat	9 (6–11)	8 (6–10)	7 (5–9)	8 (7–9)	X^2^ = 0.47, df = 2 *p =* 0.79

Almost one quarter of workers smoked daily (23% 95% CI 21–25%) and 32% (95% CI 30–34%) workers reported consuming alcohol daily. The study team observed that 11% (95% CI 10–12%) of workers appeared to be intoxicated during work. Twenty-one percent (95% CI 21–23%) of workers ate at work. At pig slaughterhouses, workers were observed to consume pieces of the carcass that were cooked over an open fire. At large mixed ruminant slaughterhouses, there was someone preparing and selling tea to workers. Twenty-four percent (95% CI 22–26%) of workers reported defecating in the open regularly.

Ninety-six percent (95% CI 95–96%) of slaughterhouse workers reported seeing the meat inspector every day (Table 3). However, only 44% (95% CI 42–46%) of workers reported the meat inspector performing antemortem inspection of the animals. Eighteen percent (95% CI 16–19%) of workers reported slaughtering animals they identified as being sick/ill/unhealthy.

Thirty-one percent (95% CI 29–33%) of the 738 slaughterhouse workers knew that disease could be transmitted from animals (Table 3). Forty-two percent (95% CI 40–44%) knew that meat could be a source of disease. Only 8% (95% CI 7–9%) of workers could name a zoonotic disease.

Eighteen percent (95% CI 16–19%) of workers reported being unwell in the past 3 months. Commonly reported symptoms included: fever (62%; 95% CI 60–64%), backache (47%; 95% CI 45–49%), headache (62%; 95% CI 60–64%), joint pain (53%; 95% CI 51–55%), cough (50%; 95% CI 48–52%), and skin infections (12%; 95% CI 11–14%).

Workers reported previous diagnoses of tuberculosis (4%; 95% CI 3–4%). Workers reported the following illnesses in the past 12 months: malaria (47%; 95% CI 45–49%); typhoid (13%; 95% CI 12–14%); respiratory illness (10%; 95% CI 9–12%); and gastrointestinal illness (4%; 95% CI 3–4%). Twenty-five percent (95% CI 23–27%) of workers reported being injured at work at least once a month and 8% (95% CI 7–9%) had a wound at the time of interview.

## Discussion

This study reports the conditions in slaughterhouses in western Kenya with respect to infrastructure, hygiene, meat inspection, and the knowledge and health of workers. The most notable findings were the lack of facilities to ensure adequate meat hygiene. Ideally the floor of the slaughterhouse should be hard concrete and impervious, to reduce dirt in the slaughterhouse and allow drainage and ease of cleaning [28]. Similarly, a roof is important to protect the carcass from the weather and to reduce the temperature in the slaughterhouse [7, 28]. Ten percent of the 142 slaughterhouses did not have a cement floor and over 30% of slaughterhouses did not have a roof.

Ideally, there should be a division in the slaughterhouse between the dirty (killing, bleeding) and clean (eviscerating and splitting) operations to prevent carcass contamination [29]. All slaughterhouses in the study area performed “batch slaughtering”. This is where an animal is killed, bled, skinned, eviscerated, and split in the same spot [8]. In the majority of slaughterhouses, carcass preparation was performed on the ground as can be observed in Fig. 3. These processes can lead to carcass contamination from the skin, the intestines and the ground [8].

International guidelines specify that hot and cold water should be readily accessible for cleaning, and that equipment and workers’ hands should be washed with soap and hot water [29, 30]. This process requires piped water facilities that are only available in a few (3%) slaughterhouses. There was a lack of water, hand washing facilities, and soap in all slaughterhouse types. Hand washing is predominantly used to protect meat from contamination, but also protects workers against directly transmitted bacterial pathogens such as *Salmonella* sp [31–33]. The lack of hand washing facilities in the majority of slaughterhouses in western Kenya has public health implications to workers and the wider community.

Only 60% of slaughterhouses had access to a toilet, with 24% of workers admitting to regularly defecating in the open. This behaviour may promote the persistence of zoonotic diseases such as cysticercosis [7]. The presence of pests and roaming animals in the slaughterhouse may contribute to infectious disease transmission, either through contamination of meat or eating of meat scraps by dogs or rats, which can lead to persistence and spread of diseases such as echinococcosis and leptospirosis [7, 28, 31].

The purpose of protective clothing within the slaughterhouse is primarily to protect the meat product from contamination but has also been shown to be protect meat handlers against directly transmitted zoonoses including leptospirosis and brucellosis [19, 30, 31]. Less than 50% of workers wore protective equipment at all times. It is likely that the cost of protective clothing is the limiting factor as the majority of workers must provide their own protective clothing. The average payment for slaughtering a single cow was reported to be US$1.10 and the cost of boots and apron US$9.50 and US$5.00, respectively.

Many of the activities in the slaughterhouses occurred without a meat inspector present, which is in violation of the stipulations in the Meat Control Act [9]. In developed countries such as the USA and the UK a licensed inspector must perform antemortem and post mortem inspection and must be present when slaughtering is being conducted for meat intended for commercial purposes [34, 35]. The USA allows ‘custom’ slaughter and the UK ‘home’ slaughter for personal consumption and this meat is not required to be inspected. All the slaughterhouses in this study sell meat to consumers and hence require inspection. It is apparent that much of the meat inspection occurs at the butchery. This hinders both antemortem inspection, which is essential for preventing the slaughter of sick animals, and detailed carcass and organ examination for signs of disease. Almost one in five workers admitted to slaughtering sick animals. Slaughtering infected animals has been shown to be a risk factor for infection with certain directly transmitted zoonotic diseases including anthrax, brucellosis, and leptospirosis [20, 31, 36, 37]. The number of inspectors may limit the amount of antemortem inspection. Meat inspectors are trained and provided by the government of Kenya. Currently inspectors attend more than 5 slaughter facilities per day. It is likely that resource restrictions limit the number and mobilisation of inspectors.

A lack of knowledge regarding the process of meat contamination is the biggest hindrance to improving conditions in the meat industry [7]. This study has shown that few people were able to name a zoonotic disease. Training personnel in meat hygiene is essential to improving conditions in slaughterhouses and to reduce bacterial contamination of meat and disease exposure in workers [8, 38].

Slaughterhouse workers have been identified in occupational health studies for elevated risk of injury, particularly to the upper extremities (mostly due to lacerations) and back injuries [39, 40]. Both backache and wounds were reported by workers. This trend may be the result of poor work practices and training or a lack of appropriate equipment [40, 41]. A large number of workers consumed alcohol regularly and over 10% appeared intoxicated at work. Alcohol consumption is a risk factor for injury at work [42].

Several potential risk factors that have been associated with zoonotic disease exposure in slaughterhouse workers in previous studies were observed in the study population. These included cutting animals throats which has been associated with RVF and brucellosis exposure [20, 43] and cleaning animal parts which is associated with brucellosis exposure [20]. Workers did not wear special protective clothing or gloves to reduce their exposure. Smoking and consuming food at the slaughterhouse have been associated with increased risk of zoonotic diseases such as leptospirosis [32]. In this study over 20% of workers smoke and ate at the slaughterhouse. The risks associated with zoonotic disease exposure will be the subject of a subsequent paper (Cook, et al.: Predictors for Rift Valley fever seroprevalence in high risk groups in western Kenya, in preparation).

Ill workers are a risk to meat contamination and should self report [7]. However, as workers are paid per animal slaughtered they are unlikely to take time off if they are feeling sick. A number of workers reported coughs, gastrointestinal and skin infections within the past 3 months. These conditions can lead to pathogen contamination of meat [7].

The majority of workers own livestock and a number of workers have a second occupation, predominantly in other aspects of the meat production industry, including as farmers or butchers, and are therefore exposed to animals and meat products outside the slaughterhouse. This increased exposure may act as a source of infection or a potential for dissemination since these activities are independently associated with disease exposure.

During the course of the study in 2012, some changes were noticed in slaughterhouses as the new Act was brought into effect. The changes were initially focused on mixed ruminant and cattle slaughterhouses, which may explain the significant difference between mixed ruminant/cattle and pig slaughterhouses documented in this study through the chi squared analysis. The lack of regulation of the pig slaughterhouses is likely to have significant public health impacts in this region particularly regarding food-borne zoonotic diseases such as cysticercosis. The prevalence of cysticercosis in pigs at slaughter in this region has been reported to be 37.6%, of which none were detected by regular meat inspection (Thomas, 2016). Potential emerging zoonotic diseases risks to pig slaughterhouse workers include *Streptococcus suis*, Methicillin-resistant *Staphylococcus aureus* and Hepatitis E [44–46].

Meat inspectors and Sub-county Veterinary Officers informed the study team that each slaughterhouse was urged to adopt one change in the first year or face closure. They were concerned that strictly enforcing the new standards would lead to a deficit in the meat industry in the region. A number of facilities, which did not make efforts to adopt the changes were closed through 2012. The informal meat industry was very difficult to quantify as slaughterhouse owners and butchers were unwilling to admit to slaughtering without authority as they feared prosecution from the public health department. This fear may explain the number of slaughterhouses that declined to participate. Despite only 2 slaughterhouses being classified as category B, a large number of slaughterhouses were trading meat beyond the local village where the slaughter was conducted which contravenes the regulations of the Meat Control Act and may allow the dissemination of disease.

Fear of stigma associated with certain disease diagnoses such as HIV may have influenced participation in the study. The incentive of free treatment for diagnosed conditions and the high response rate (73.4%) should reduce response bias. In addition the questionnaires were pretested to identify any problems with the data collection tools before the study and observational data regarding facilities at the slaughterhouses was collected to confirm that responses to the foreman questionnaire were accurate.

The findings of this study are similar to reports from other countries in East Africa regarding lack of facilities, hygiene, and inadequate meat inspection [15, 18]. These findings are likely to be indicative of slaughterhouses in rural areas across the region. As other authors have suggested training workers and inspectors in hygiene practices and improving infrastructure are likely to reduce meat contamination and dissemination of disease. Training should focus on clean evisceration, hand washing, instrument washing, carcass trimming, protective equipment [16, 17] and inspection [12, 18].

Slaughterhouse workers may act as sentinels for disease outbreaks in animals and people [43, 47]. This study did not measure spccific disease risks but a number of previously reported risk factors were identified, highlighting the potential for slaughterhouse workers to be exposed to disease. An increase in febrile illness in this occupational group could indicate an outbreak of an emerging or reemerging pathogen in animals. Health care workers should be educated to the increased risks for illness and injury faced by these workers and be alerted to the potential that disease issues in slaughterhouse workers might indicate an increase in these conditions in animals. Targeting slaughterhouse workers through enhanced surveillance might be a cost-effective method to detect disease outbreaks in animals and people in a community. Surveillance tools might involve targeted sampling in a selection of slaughterhouse facilities or raising an alert when a slaughterhouse worker reports to a health unit. This “One Health” approach to disease surveillance is likely to benefit workers, producers and consumers.

## Conclusion

This study contributes to understanding the current situation in the meat industry in western Kenya. The study documents the conditions in slaughterhouses in western Kenya during the implementation of the revised Meat Control Act, 2012 and gives an indication where improvements need to be made. None of the slaughterhouses visited complied with the published regulations at the time of the study, with many falling far below a minimum standard. The infrastructure at the majority of slaughterhouses did not meet the guidelines with many slaughterhouses lacking basic structural requirements such as a roof and sanitation facilities such as a toilet or running water.

The results of this study are an important contribution to understanding and regulating the meat industry in Kenya and it is likely that they can be generalised to rural areas in other resource limited settings. Improvements need to be made to facilities and practices in all slaughterhouses. In the initial stages, training is recommended to improve awareness for workers, managers, and inspectors of the risks of meat contamination and methods to reduce it. Secondly, improvement of facilities must be implemented with closure of sub-standard facilities and focusing resources on fewer facilities to improve meat hygiene in this resource-limited setting. These enforcements would need to take into consideration the effects on the price of meat and nutrition in the region and the risk of pushing meat producers into the informal market.

## Additional files

Additional file 1: Slaughterhouse worker questionnaire. A transcript of the questionnaire administered to individual slaughterhouse workers outlining the quesitons regarding personal history, knowledge of zoonoses, risk behaviours, exposure to livestock, and personal hygiene practices at the slaughterhouse. (DOCX 110 kb)
Additional file 2: Slaughterhouse foreperson questionnaire. A transcript of the questionnaire administered to the slaughterhouse foreperson regarding slaughterhouse structure, equipment, and practices. (DOCX 80 kb)

## Abbreviations

CI: Confidence interval
FAO: Food and Agriculture Organization of the United Nations
GIS: Geographical information systems
GPS: Global Positioning System
ILRI: International Livestock Research Institute
KEMRI: Kenya Medical Research Institute
MRC: Medical Research Council
PAZ: People, Animals and their Zoonoses
PDA: Personal digital assistant
RVF: Rift Valley fever
