# Supplementary material for: Working conditions and public health risks in slaughterhouses in western Kenya
Source: BMC Public Health. 2017 Jan 5;17:14. doi: 10.1186/s12889-016-3923-y (PMC5217581; doi:10.1186/s12889-016-3923-y)
Supplement: Additional file 1: — Slaughterhouse worker questionnaire. A transcript of the questionnaire administered to individual slaughterhouse workers outlining the quesitons regarding personal history, knowledge of zoonoses, risk behaviours, exposure to livestock, and personal hygiene practices at the slaughterhouse. (DOCX 110 kb) [file 12889_2016_3923_MOESM1_ESM.docx]

## Slaughterhouse individual questionnaire

**General**

1. Date --/--/----
2. Start time --:--
3. Recorder <name> (look up list)
4. Slaughterhouse barcode <number> Scan
5. Respondent age: -- (in age groups – as people do not know exact age)
6. Respondent sex: Male / Female
   1. If female are you pregnant? Y/N/NR
      1. If pregnant What stage? 1^st^, 2^nd^, 3^rd^ trimester
7. Does this participant meet the selection requirement (over 18) and given informed consent? Yes / No (Terminate)
8. Interviewee barcode SCAN <number>
9. Language of questionnaire administration (Language look up)

Teso; Samia; Bukusu; Luhya; Luo; Swahili; English; Kamba; Kalenjin; Other

1. Tribal origin (Tribe look up)

Teso; Luhya; Luo; Kikuyu; Samia; Saboat; European; Kamba; Kalenjin; Other

1. Principal religion (religion look up)

Roman catholic; Protestant; Other Christian; Muslim; Traditional religion; None; Other; NR

1. Marital status (marital look up)

Single; Married; Divorced; Widowed; NR

1. Do you have children? Y/N/NR
   1. If yes, how many living? (popup table – numbers up to 30)
2. Have you lived outside this province at any time for more than 6 months? Y/N/NR
   1. Where did you live? Popup list provinces
3. How many people live in your homestead? (popup list numbers)
4. How many rooms in your homestead? (popup list numbers)
5. What level of education have you reached? (look up education)

No formal education; Pre-school; Primary; Secondary; Tertiary; College; University; Vocational/technical school; Other; NR

1. Do any members of your homestead work in healthcare? Y/N/NR
   1. What do they do? (look up healthcare)

Doctor, nurse, midwife, clinical officer, nursing assistant, admin, cleaner, security, NR. other

1. Do you have contact with livestock outside of work? Y/N/NR
   1. Which ones? (multiselection list)

Cattle, sheep, goats, pigs, poultry, rabbits, NR, Other

1. Do you or your family keep livestock at you normal place of residence? Y/N/NR
   1. Which ones? (multiselection list)

Cattle, sheep, goats, pigs, poultry, rabbits, NR, Other

1. Do any members of your homestead work in livestock farming? Y/N/NR
   1. What animals?

Cattle, sheep, goats, pigs, poultry, rabbits, NR, Other

1. Do you have contact with dogs? (look up freq)

No; Daily; At least once a week; At least once per month; At least once per year; Used to but no longer; NR

1. In the last 12 months have you been hunting? (look up freq)

No; Daily; At least once a week; At least once per month; At least once per year; Used to but no longer; NR

**Food preferences**

1. Do you eat beef? Y/N/NR
   1. How often do you eat beef? (look up freq)

Never; Daily; At least once a week; At least once per month; At least once per year; Only on special occasions; Used to but no longer; NR

1. Do you eat pork? Y/N/NR
   1. How often do you eat pork? (look up freq)

Never; Daily; At least once a week; At least once per month; At least once per year; Only on special occasions; Used to but no longer; ND

1. In the last 12m have you drunk cow’s milk? Yes No NR
   1. How do you take your milk? (multiselection)

Boiled; Soured; Raw; Pasteurised; Other (allow all answers)

1. In the last 12m have you drunk goat’s milk? Yes No NR
   1. How do you take your milk? (multiselection)

Boiled; Soured; Raw; Pasteurised; Other

1. Do you take animal blood? Yes No NR
   1. How do you take animal blood? (multiselection)

Boiled, Cooked, Raw, Other

1. Do you smoke cigarettes? (look up table freq)

No; Daily; At least once a week; At least once per month; At least once per year; Used to but no longer; NR

- 1. If daily or weekly what is the number of cigarettes you smoke per week? <number>

1. Do you consume alcohol? (look up freq)

No; Daily; At least once a week; At least once per month; At least once per year; Used to but no longer; NR

**Sanitation**

1. Where do you obtain water (for personal use)? (look up water)

Private borehole; River; Shared borehole; Municipal water (tap); Well; Spring; Dam; Pond: Other; Lake; NR

1. How often do you use the latrine when you need to defecate? (look up defecation)

Everytime; Mostly; Sometimes; Rarely; Never; NR

**Health status**

1. Have you had a period of illness in the past 12 months?

Yes No NR

- 1. If you have had a health problem, please list <open>

1. Have you had fever in the last 3 months? Y/N/NR
2. Have you had headache in the last 3 months? Y/N/NR
3. Have you had backache in the last 3 months? Y/N/NR
4. Have you had joint pain in the last 3 months? Y/N/NR
5. Have you had seizures in the last 3 months? Y/N/NR
6. Have you had weightloss in the last 3 months? Y/N/NR
7. Have you had cough in the last 3 months? Y/N/NR
8. Have you had nausea in the last 3 months? Y/N/NR
9. Have you had vomiting in the last 3 months? Y/N/NR
10. Have you had diarrhoea in the last 3 months? Y/N/NR
11. Have you had loss of appetite in the last 3 months? Y/N/NR
12. Have you had abdominal pain in the last 3 months? Y/N/NR
13. Have you had a skin infection in the last 3 months? Y/N/NR
14. Have you had any boil in the last 3 months? Y/N/NR
15. Have you taken any medicines in the last 3months

Yes No Don’t know NR

- 1. If you have taken medications in the last month, tick all that apply

Unknown; Chloroquine; Other antimalarial; Anti-retroviral; Antibiotic; Anti-tussive; Rehydration solution; Anti-inflammatory or pain killer; Heart medication; Insulin; Medication for seizures; Dewormers; Other; ND

1. Have you used any antimicrobial cream or ointment in the last 3 months? Y/N/NR
2. Usually, when you feel ill, where do you seek treatment? (look up treatment)

Don’t seek treatment; Family member; Community health worker; Traditional healer; Chemist; Private clinic; Hospital; Self treatment; Neighbour; Church healer; Other; NR

1. Have you visited a clinic, community health centre or doctor in the last 3 months? Y/N/NR
2. Have you visited a hospital in the last 3 months? Y/N/NR
   1. Have you been admitted (stayed overnight) to a hospital in the last 3months? Y/N/NR
3. Are you aware of ever having brucellosis?

Yes No Don’t know NR

1. Are you aware of ever having tuberculosis?

Yes No Don’t know NR

1. Are you aware of ever having q fever?

Yes No Don’t know NR

1. Are you aware of ever having tapeworm?

Yes No Don’t know NR

1. Are you aware of ever having Rift Valley fever?

Yes No Don’t know NR

**Knowledge of food-borne and zoonotic disease**

1. Are you aware of any disease you might catch from contact with animals?

Yes No Don’t know NR

- 1. Which symptoms/disease might you catch from animals? (multiselection)

Unknown disease; Malaria; Fever; Stomach pain; Diarrhoea; Respiratory difficulties; Fever; Skin rash; Seizures; Brucellosis; Anthrax; TB; RVF; Q fever; Sleeping sickness; Tapeworm; Leptospirosis; Salmonella; E.coli; Rabies; Toxoplasma; Other; NR

If answered other please type________

1. Are you aware of any diseases that you might catch from eating meat? Y N DK NR
   1. Which symptoms/disease might you catch from eating meat (multiselection)

Unknown disease; Malaria; Fever; Stomach pain; Diarrhoea; Respiratory difficulties; Fever; Skin rash; Seizures; Brucellosis; Anthrax; TB; RVF; Q fever; Sleeping sickness; Tapeworm; Leptospirosis; Salmonella; E.coli; Rabies; Toxoplasma; Other; NR

If answered other please type________

**Slaughterhouse questions**

1. How long have you been a slaughterhouse worker? <number years>
2. How long have you worked in this slaughterhouse? <number years>
3. Do you work in another slaughterhouse at present? Yes No NR
4. Which other SH? ___________
5. How many days per week do you work as a slaughterhouse worker? 1 2 3 4 5 6 7
6. How many hours per day do you work? 1 2 3 4 5 6 7 8 9 10 11 12
7. What is your occupation in the SH? (look up Job in SH)

Slaughterman; Flayer; SH owner; Butchery owner; Clean the intestines; Cleaner; Other: NR

1. Do you have another occupation? Yes /No/NR
2. What is this occupation? (popup Y/N/NR)
   1. What is this occupation? (look up occupation)

Meat business owner; Farmer; Trader; Shop keeper; Student; Driver; Butcher; Fisherman; Boda driver; Carpenter; Mason; Taxi driver; Other; NR

If other enter details:

1. Are you involved directly in slaughter/flaying? Y/N/NR
   1. How many animals do you personally slaughter a day on average? <number>
   2. Who provides you equipment? Look up list

Butcher; Meat inspector; SH owner; Worker; Other; NR

- 1. Is your equipment used exclusively within the slaughterhouse? Y N DK
  2. How often do you clean your equipment? (look up wash freq)

Between animals, Before slaughtering; After slaughtering; Daily; Weekly; Never; NR

- 1. What do you use to clean your equipment? (multilist cleaning)

Water; Bleach; Ammonia; Soap; Washing powder; Nothing; NR

- 1. How often do you or someone else sharpen your equipment? (look up freq)

Daily; Weekly; Monthly; Never; NR

- 1. Have you been for a medical check up recently (in the last 6 months)? Y/N/NR
  2. Do you have a license for slaughtering? Y/N/NR

1. Do you wear protective clothing eg. Coveralls, overalls, apron? Always/Sometimes/Never
   1. What protective clothing? (Look up clothing)

Coveralls, Overalls, Apron, Lab coat, Other. NR

- 1. Who provides?

Meat inspector; Butcher; Worker; SH owner; Other; NR

1. Do you change clothes when you leave the slaughterhouse?

Yes No NR

1. Where do you wash your clothes/shoes after slaughtering?

River; Home; At the slab; Give to someone; Other; NR

1. Do you wear footwear in the slaughterhouse? Y/N/NR

Always/Sometimes/Never

- 1. What type?

Sandals, Boots, Shoes, Rubber boots, Running shoes, Other, NR

- 1. Who provides?

Meat inspector; Butcher; Worker; SH owner; Other; NR

1. Do you wear gloves when slaughtering? Always/Sometimes/Never
2. When do you wash your hands? (tick all that apply) (multiselection)

Before slaughtering; After slaughtering; Between animals; Before I go home; After I use the latrine; NR

1. Is there soap provided for hand washing?

Always/Sometimes/Never

1. Do you injure yourself at work and how often? (look up freq)

Daily, Weekly, Monthly, Never

1. Do you eat at the slaughterhouse? (Look up meat inspector freg)

Every time we slaughter; Most times(once a week); Sometimes (once a month); Rarely (once a year); Never; NR; DK

1. What would you do with a sick animal? (look up sick animal)

Send home; slaughter last and condemn; slaughter and sell; slaughter and keep for own consumption, Treat, Ask doctor, Slaughter and ask doctor, Other, NR

1. What would you do with an animal that dies on the way or at the slaughterhouse?

Send home; slaughter last and condemn; slaughter and sell; slaughter and keep for own consumption, Treat, Ask doctor, Slaughter and ask doctor, Other, NR

1. What animals do you slaughter/flay/clean?

Cattle only, Sheep/goats only; Cattle and Sheep/goats; Pig only; Pigs and cattle; Pigs and sheep/goats; NR

IF cattle

1. Have you seen these lesions (show picture of tuberculous lung/liver)? Pictures are available in appendix 7

Yes; No; Don’t know; NR

If yes:

- 1. How often? (look up freq)

Daily; Weekly; Monthly; Yearly; DK

- 1. Named correctly Yes; No;
  2. What do you do with these animals? (look up organ disposal)

Slaughter as normal, Dispose of entire carcass, Remove affected organs

1. Have you seen these lesions (show picture of brucellosis lesions)?

Yes; No; Don’t know; NR

If yes:

1. How often? (look up freq)

Daily; Weekly; Monthly; Yearly; DK

1. Named correctly Yes; No;
2. What do you do with these animals? (look up organ disposal)

Slaughter as normal, Dispose of, Remove affected organs

1. Have you seen these lesions (show picture of skin lesions on people)?

Yes; No; Don’t know; NR

If yes:

1. How often? (look up freq)

Daily; Weekly; Monthly; Yearly; DK

1. Named correctly Yes; No;
2. Have you seen these lesions (show picture of Echinococcus)?

Yes; No; Don’t know; NR

If yes:

1. How often? (look up freq)

Daily; Weekly; Monthly; Yearly; DK

1. Named correctly Yes; No;
2. What do you do with these animals? (look up organ disposal)

Slaughter as normal, Dispose of, Remove affected organs

If pigs

1. Have you seen these lesions (show picture of *Taenia* cysts)?

Yes; No; Don’t know; NR

If yes:

1. How often? (look up freq)

Daily; Weekly; Monthly; Yearly; DK

1. Named correctly Yes; No;
2. What do you do with these animals? (look up organ disposal)

Slaughter as normal, Dispose of, Remove affected organs

1. How often does the meat inspector visit? (look up freq)

Every time we slaughter; Most times (once a week); Sometimes (once a month); Rarely (once a year); Never; NR; DK

1. Does the meat inspector examine the animals before they are slaughtered?

Always, Sometimes, Never, NR

- 1. Does he/she ever refuse the slaughtering of an animal?

Yes/No/NR

- 1. How often does the meat inspector refuse to allow an animal to be slaughtered?

Every time we slaughter; Most times (once a week); Sometimes (once a month); Rarely (once a year); Never; NR; DK

- 1. For what reason would the meat inspector refuse slaughter? Multilist

Sickness, Diarrhoea, Coughing, Injury, Emaciation, Death. Other. DK, NR

1. How often does the meat inspector ever condemn animal or part of an animal (organ)? (look up freq)

Every time we slaughter; Most times (once a week); Sometimes (once a month); Rarely (once a year); Never; NR; DK

- 1. Which parts? Liver, Kidney, Heart. Lung, Intestines, Muscle
  2. What happens to these organs? (look up organ disposal)

Pit, Dog, Home

1. Does the meat inspector ever condemn an entire carcass? Y/N/NR
   1. How often?

Every time we slaughter; Most times (once a week); Sometimes (once a month); Rarely (once a year); Never; NR; DK

- 1. What happens to the carcass?

Pit, Dog, Home

1. In the last 12 months have you seen rats around the slaughterhouse? (look up freq)

No; Daily; At least once a week; At least once per month; At least once per year; Used to but no longer; NR

1. In the last 12 months have you seen wildlife around the slaughterhouse? (look up freq)

No; Daily; At least once a week; At least once per month; At least once per year; Used to but no longer; NR

- 1. What wildlife have you seen? (multiselection list)

Bats, rodents, Mongoose, birds, Snakes, lizards, monkeys, other, NR

**Observational**

1. Weight
2. Height
3. Midupper Arm Circumference
4. Temperature
5. Splenomegaly
6. Hepatomegaly
7. Abdominal distension
8. Membrane pallor
9. Jaundice
10. Limb oedema
11. Rash
12. Wounds requiring treatment
13. Does the individual have a BCG scar?
14. Does the worker appear drunk?
15. Red top barcode
16. Purple top bar code
17. Was a stool sample collected?
    1. Barcode
18. Was a stool swab sample collected?
    1. barcode
19. Was a sputum sample collected?
    1. barcode
20. Was a nasal swab sample collected?
    1. barcode
21. Thick film barcode
22. Thin film barcode
