# Supplementary material for: Working conditions and public health risks in slaughterhouses in western Kenya
Source: BMC Public Health. 2017 Jan 5;17:14. doi: 10.1186/s12889-016-3923-y (PMC5217581; doi:10.1186/s12889-016-3923-y)
Supplement: Additional file 2: — Slaughterhouse foreperson questionnaire. A transcript of the questionnaire administered to the slaughterhouse foreperson regarding slaughterhouse structure, equipment, and practices. (DOCX 80 kb) [file 12889_2016_3923_MOESM2_ESM.docx]

## Slaughterhouse foremen questionnaire

1. Date --/--/----
2. Start time --:--
3. Recorder <name>
4. Slaughterhouse barcode <number>
5. Sex of respondent
6. Language of questionnaire
7. Job in slaughterhouse
8. How many slaughter men
9. How many are flayers
10. How many are licensed
11. Which animals are slaughtered?

Cattle only, Cattle and sheep/goats, Pigs only, Pigs and cattle, Pigs and sheep/goats.

If cattle/sheep/goats

Number of cattle slaughtered per week (average)

How are the cattle transported?

Number of sheep/goats slaughtered per week (average)

How are the sheep/goats transported?

If pigs

Number of pigs slaughtered per week (average)

How are the pigs transported?

1. Where is the meat from this slaughterhouse sold?

Locally

Exported to other districts

1. How is the meat transported?
2. What would you do with a sick animal?
3. What would you do with a dead animal?
4. How often does the meat inspector visit the slaughterhouse?

Daily Weekly Fortnightly Monthly

1. Does the meat inspector examine animals before slaughter?
2. Does the meat inspector refuse to allow slaughter?
3. For what reasons does he refuse to allow slaughter?
4. How often?
5. Does the meat inspector condemn organs?
6. How often does the meat inspector condemn organs?
7. For what reason does the meat inspector condemn organs?
8. What organs does the meat inspector most commonly condemn?
9. Does the meat inspector condemn the entire carcass?
10. How often does the meat inspector condemn the carcass?
11. For what reason does the meat inspector condemn the carcass?
12. What method of euthanasia do you employ?
13. Is specialized protective clothing worn whilst in the slaughterhouse?

Y N DK

1. Type: Overalls, Apron, Lab coat
2. Who provides this clothing?
3. Is this clothing worn exclusively in the slaughterhouse?
4. Where is this clothing/footwear cleaned/laundered?

At the slab, at individual homes,

1. Is specialized protective footwear worn whilst in the slaughterhouse?

Y N DK

1. Type Boots, Closed shoes, Slippers
2. Is this provided by the SH? Y N DK
3. What specialized equipment is used within the slaughterhouse?

Winch; Trolley; Saw; knives; Axe

1. How often is the equipment cleaned?

Between animals, Before slaughtering; After slaughtering; Daily; Weekly; Never; NR

1. What do you use to clean your equipment?

Water; Bleach; Ammonia; Soap; Nothing; NR

1. Is this equipment used exclusively within the slaughterhouse? Y N DK
2. How often do you clean the slab?

Between animals, Before slaughtering; After slaughtering; Daily; Weekly; Never; NR

1. What do you use to clean the slab?

Water; Bleach; Ammonia; Soap; Nothing; NR

1. Do dogs come to the slaughterhouse?

Never; Daily; At least once a week; At least once per month; At least once per year; Used to but no longer; ND

- 1. Do you feed the dogs internal organs of animals?

Always Sometimes Never

1. Do cats come to the slaughterhouse?

Never; Daily; At least once a week; At least once per month; At least once per year; Used to but no longer; ND

- 1. Do you feed the cats internal organs of animals?

Always Sometimes Never

1. What disposal method is employed for carcass waste?

Pit; Bury; Throw away; Feed to dogs; Sell; Take home; NR

1. What disposal method is employed for condemned carcasses?

Pit; Bury; Throw away; Feed to dogs; Sell; Take home; NR

1. Where do you obtain your water for slaughtering?

Borehole; River; Pump; Tap; Well; Spring; Other; NR

1. Is there a place for hand washing? Y/N
2. Is soap provided for hand washing? Y/N
3. Is there a latrine? Y/N
4. In the last 12 months have you seen rats around the slaughterhouse?

No; Daily; At least once a week; At least once per month; At least once per year; Used to but no longer; ND

1. In the last 12 months have you seen wildlife around the slaughterhouse?

No; Daily; At least once a week; At least once per month; At least once per year; Used to but no longer; ND

1. What wildlife have you seen around the slaughterhouse?

Bushbuck; Other bovid; Bats; Mongoose; Snake; Monitor lizard; Monkey; Wild birds; Other

1. Structure of slaughterhouse floor

Earth Cement Tile Timber

1. Structure of sides

No sides Timber Unburnt bricks Burnt bricks Stone Cement Mud

1. Structure of roof

No roof Tile Thatch Iron sheets

1. Is there electricity in the slaughterhouse? Y/N
2. How is water access?
3. Is there a latrine in the compound?
4. Type of latrine

Latrine completely closed Partially closed Open pit

1. Evidence of latrine use
2. Evidence of animal scavenging around the latrine
3. Did you see workers wearing PPE>
4. Type?
5. Did you see workers wearing shoes?
6. Type
7. Did you see workers wearing gloves?
8. Name of market
9. GPS Northing
10. GPS Easting
11. Altitude
12. GPS code
